# Characterization of Negative Feedback Network Motifs in the TGF-β Signaling Pathway

**DOI:** 10.1371/journal.pone.0083531

**Published:** 2013-12-20

**Authors:** Daniel Nicklas, Leonor Saiz

**Affiliations:** Modeling of Biological Networks Laboratory, Department of Biomedical Engineering, University of California Davis, Davis, California, United States of America; University Medical Center Freiburg, Germany

## Abstract

{Chung, 2009 #1}The transforming growth factor-β (TGF-β) superfamily of cytokines plays a fundamental role in a wide variety of cellular processes, including growth, differentiation, apoptosis, and tissue homeostasis. Its relevance is emphasized by the mutations of its core components that are associated with diverse human diseases, such as cancer and cardiovascular pathologies. A prominent regulator of the pathway is Smad7, which attenuates the signal and controls its duration in a cell-type-dependent manner through a negative feedback loop. Here, we characterize all the potential Smad7-mediated negative feedback network motifs and investigate their effects on the signaling dynamics upon stimulation with TGF-β and bone morphogenetic protein (BMP) ligands. The results show that the specific negative feedback implementation is a key determinant of both the response of the system to single and multiple ligands of the TGF-β superfamily and its robustness and sensitivity to parameter perturbations.

## Introduction

The transforming growth factor-β (TGF-β) superfamily, which comprises 33 different ligands in mammalian cells, plays a fundamental role in development and maintenance of tissue homeostasis [1]. These ligands regulate key cellular processes, such as proliferation, motility, differentiation, and apoptosis [2]. Dysregulation of the TGF-β signal transduction pathway resulting from mutations of its core components has been associated with a number of human diseases, including cancer and vascular disorders [3], [4]. As a result, a significant effort of clinical research focuses on developing therapies targeting the TGF-β pathway [1].

The multiple cellular effects elicited by the TGF-β superfamily ligands are triggered by binding of the ligand to two types of receptor serine/threonine protein kinases (type II and type I receptors) at the plasma membrane, which then form an active ligand-receptor complex. The signal is thenceforth propagated through the intracellular Smad proteins into the nucleus where activated Smad complexes act as transcription factors, controlling the expression of hundreds of genes in a cell-type and context dependent way [2]. Specifically, the active ligand-receptor complex is internalized into early endosomes, where it recruits and phosphorylates one of the receptor-regulated Smad (R-Smad) proteins. Phosphorylated R-Smads bind Smad4, the common-mediator Smad, forming a heterooligomer that translocates into the nucleus and binds to DNA to regulate the expression of its target genes. Ligands of the TGF-β superfamily signal through the activation of two parallel R-Smad channels. Specifically, bone morphogenetic proteins (BMPs) activate the Smad1/5/8 channel; nodal and activin ligands activate the Smad2/3 channel; and TGF-β activates both channels [5].

Inhibitory Smads (I-Smads), Smad6 and Smad7, negatively regulate signaling in this pathway, antagonizing the effects of R-Smads [6]. They inhibit the signal through different mechanisms, such as sequestering phosphorylated R-Smads, specifically Smad1, in an inactive complex as observed for Smad6 [7] or, more typically, by competing with R-Smads for receptor binding [8], [9] and promoting degradation of ligand-receptor complexes through Smurf-dependent ubiquitination [10], [11]. Importantly, this inhibition can occur through a negative feedback loop because TGF-β superfamily ligands induce transcription of *Smad6* and *Smad7* genes by the binding of nuclear phosphorylated R-Smad–Smad4 complexes to the promoters [1].

In the last few years, several mathematical models of the Smad-dependent TGF-β signal transduction pathway have been developed to get insights into its functioning [12]–[25]. In particular, a few of these computational models have incorporated the Smad7-mediated negative feedback loop as an explicit component of the pathway in order to investigate its mechanistic role in the observed behavior [12], [15], [16], [24], [25]. In these cases, the effects of Smad6, which preferentially blocks BMP signaling, are typically combined with those of Smad7, which blocks both TGF-β and BMP signaling, in a single effective inhibitory component. We have previously demonstrated that differences in the implementation of the negative feedback loop capture the distinct signaling dynamics of diverse cell lines [25]. In addition to investigating the dynamic response of TGF-β signaling, quantitative approaches have revealed how the signaling behavior is affected by perturbations of its parameters through the use of sensitivity analyses [12]–[15], [17], [22], [25], analytical calculations [18], and other types of mathematical analysis [19].

In mammalian cells, the Smad7-dependent negative feedback loop has different implementations in different cell lines [25]. For example, bovine aortic endothelial cells (BAECs) exhibit an auto-regulatory negative feedback loop, where Smad7 is expressed through activation of the Smad1 channel and inhibits further activation of the same R-Smad channel [26]. The mouse myoblast cell line C2C12 displays a cross-regulatory negative feedback, where Smad7 is expressed through activation of the Smad2 channel, but inhibits the Smad1 channel when the pathway is stimulated with TGF-β [25], [27], [28]. Human keratinocytes HaCaT cells exhibit a high basal concentration of Smad7 that is minimally affected upon treatment with TGF-β [29].

Robustness is a fundamental characteristic of biological systems, defining their ability to maintain normal function despite perturbations of their components [30]. In the TGF-β signal transduction pathway, negative feedback control in specific cases has been shown to confer robustness to the system, reducing phenotypic variability in cell populations [16], and to decrease the sensitivity of the signaling output to perturbations of its parameters [15]. A typical avenue to analyze the systemic robustness of a system is to perturb the model parameters, quantify this variation from the nominal parameter set, and assess the properties of the system output in the perturbed state. This type of approach has been used to study models of bacterial chemotaxis [31], the mitogen-activated protein kinase (MAPK) cascade [32], the interferon-gamma (IFN-γ) induced Janus kinase (JAK) signal transducers and activators of transcription (STAT) pathway [33], the B-cell lymphoma 2 (Bcl-2) apoptotic switch [34], and the epidermal growth factor receptor (EGFR) signaling network [35].

Here we characterize all the potential Smad7-mediated negative feedback network motifs of the TGF-β signaling pathway and study their effects on the signaling dynamics, robustness, and sensitivity of a detailed mathematical model of the pathway. This type of study is notably important because network motifs, defined by a particular pattern of biochemical interactions, may reflect important functional properties of the system [36]. We investigate the dynamic response by exposing the system to two different extracellular ligand conditions, namely to stimulation with TGF-β ligand alone and to co-stimulation with TGF-β and BMP ligands. We do not consider Smad6 explicitly because its effects can be taken into account effectively by changing the strength of Smad7 interactions and it does not give rise to any new negative feedback network motif. The robustness analysis considers a single measure for the whole system and provides insight into how the architecture of the network shapes the model's response to systemic parameter perturbation. Subsequently, to elucidate the effects of individual parameter perturbation on the model output, we use a global sensitivity analysis, which evaluates these effects within a large parameter space [37]. As the output of the model we focus on different properties of the nuclear concentration of phosphorylated R-Smad-Smad4 complexes, as they act as transcription factors to control the expression of the target genes. Our analysis provides a comprehensive examination into the effects of distinct negative feedback network motifs in defining the system behavior in the TGF-β signal transduction pathway.

## Methods

### TGF-β signal transduction pathway model

**Figure 1:**
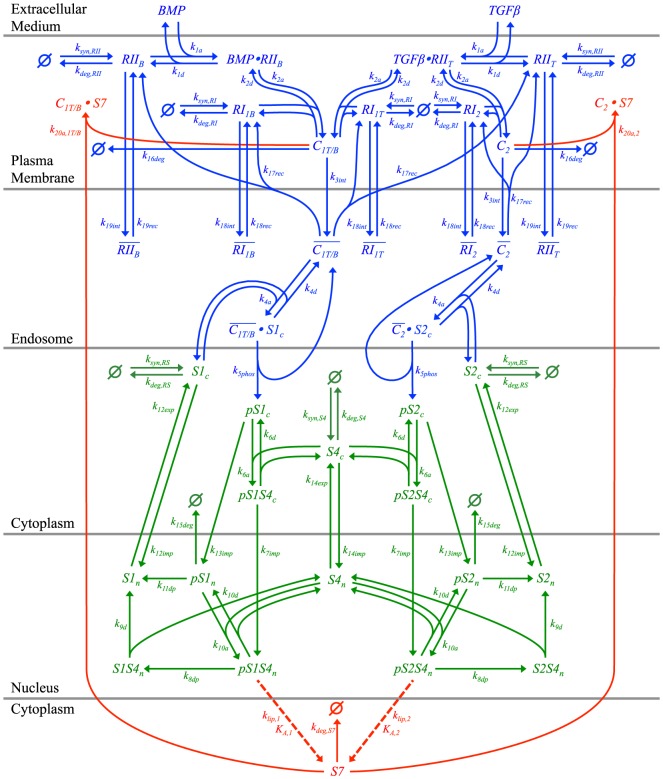
Schematic illustration of Smad-dependent TGF-β signal transduction pathway model. Arrows indicate reaction steps along the pathway and are labeled with the rate constant for the reaction. Dashed arrows denote Smad7 synthesis through gene expression. We use overbars to represent internalized receptor species in the endosome and the symbol “/” to indicate “or” in grouping the *C_1T_* and *C_1B_* ligand-receptor complexes as *C_1T/B_*. Different colors group the three modules, where blue indicates receptor trafficking, green indicates Smad nucleocytoplasmic shuttling, and red indicates negative feedback.

We consider the detailed model developed in Ref. [25] to assess the Smad-dependent response to treatment with TGF-β and BMP ligands. We have shown elsewhere [25] that this detailed computational model accurately reproduces the diverse behavior of experimental datasets for human keratinocytes (HaCaT), bovine aortic endothelial cells (BAEC), and mouse mesenchymal cells (C2C12). The model includes three modules of the signaling pathway, namely receptor trafficking, nucleocytoplasmic shuttling of two parallel R-Smad channels, and a Smad7-based negative feedback loop. Signaling is initiated when a TGF-β or BMP ligand binds to its type II receptor, denoted as *RII_T_* or *RII_B_*, respectively. This ligand-receptor complex then recruits a type I receptor, either *RI_1T_*, *RI_1B_*, or *RI_2_*. The former two type I receptors signal through the Smad1 channel after binding to TGFβ-*RII_T_* or BMP-*RII_B_* ligand-receptor complexes, respectively, while the latter signals through the Smad2 channel after binding to the TGFβ-*RII_T_* complex. The resulting active heteromeric ligand-receptor complexes are denoted by *C_1T_*, *C_1B_*, or *C_2_*, respectively, where the subscript is identical to that of the type I receptor within the complex. The active ligand-receptor complexes are then internalized into the early endosome, which provides a platform to efficiently phosphorylate cytosolic Smad1 (*S1_c_*) or Smad2 (*S2_c_*). We use the subscripts *c* and *n* to indicate cytosolic and nuclear species, respectively, and the prefix *p* to denote phosphorylated species. In the cytosol, *pS1_c_* and *pS2_c_* bind to Smad4 (*S4_c_*) to form the *pS1S4_c_* and *pS2S4_c_* complexes, respectively, which translocate into the nucleus. The complexes *pS1S4_n_* and *pS2S4_n_* then bind to DNA and activate the expression of Smad7 (*S7*), which irreversibly binds to surface ligand-receptor complexes (*C_1T_*, *C_1B_*, or *C_2_*), preventing their association with and phosphorylation of R-Smad proteins, and targeting the active ligand-receptor complexes for degradation. Therefore, the negative feedback loop is initiated with expression of Smad7 through the Smad1 and Smad2 channels by way of *pS1S4_n_* and *pS2S4_n_*, respectively. Smad7 proteins then inhibit the activation of the Smad1 channel by binding to *C_1T_* and *C_1B_*, while binding to *C_2_* inhibits Smad2 channel activation. A schematic representation of the model is shown in Figure 1, where arrows indicate each modeled reaction in the pathway. Reactions are mathematically represented with mass-action kinetics, which are then used to form the system of ordinary differential equations (ODEs) to track the change in concentration of each modeled species over time (Table 1).

**Table 1:** System of ODEs for each modeled species.

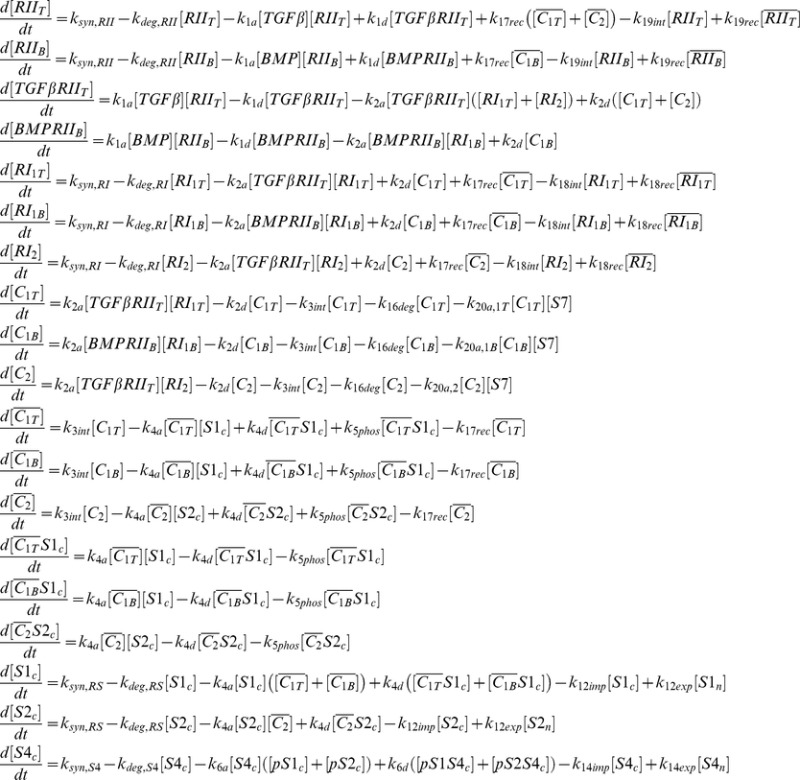	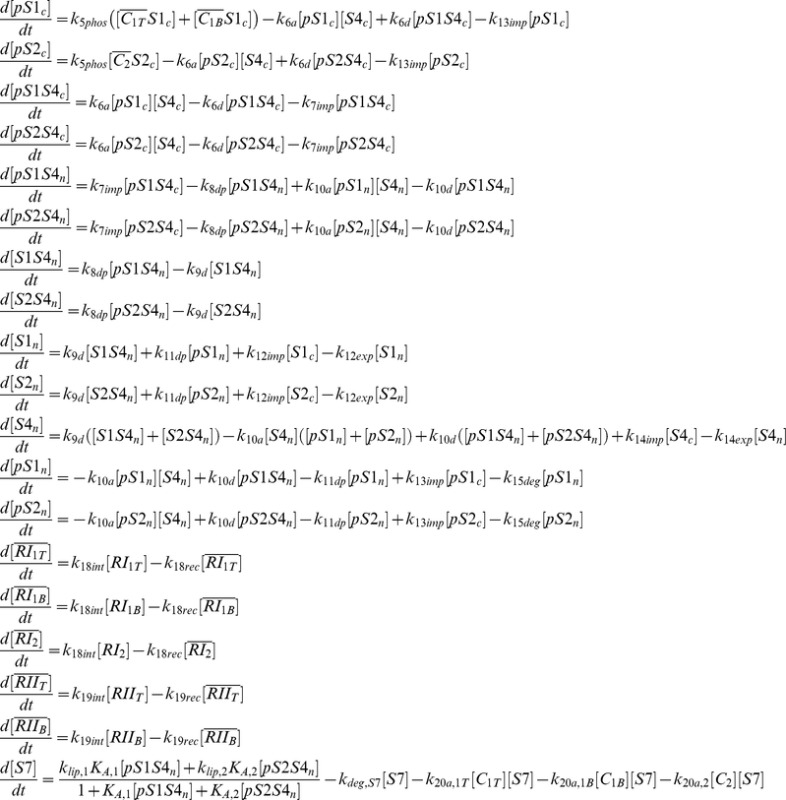

Equations track the change in concentration of each modeled species over time *t*. Overbars indicate receptor species internalized in the endosome. We prefix a species variable name with *p* to denote that it is phosphorylated and use the subscripts *c* and *n* represent cytoplasmic and nuclear species, respectively.

To define the reference parameter set for the model, we use as starting point the parameter values defined for HaCaT cells in Ref. [25]. We assume that Smad1 and Smad2 have the same abundance and reaction dynamics. For the association rate of Smad7 with *C_2_* (*k_20a,2_*), we use 1.50×10^−4^ molecules^−1^ min^−1^, which corresponds to the initial estimated value before optimization for HaCaT cells [25]. This higher affinity value provides stronger inhibition through the negative feedback loop, but does not qualitatively affect the robustness and sensitivity results (see Text S1). We then set the parameters governing Smad7 expression by, and inhibition of, the Smad1 channel, namely *k_20a,1T_, k_20a,1B_, k_lip,1_*, and *K_A,1_* to the corresponding values for the Smad2-channel-associated counterparts: *k_20a,2_, k_lip,2_*, and *K_A,2_*.

Prior to ligand stimulation, we determine the steady state solution for the system of ODEs (Table 1) by setting each time-derivative to zero and solving the linear system of equations that arises with *TGFβ* and *BMP* equal to zero, the pre-stimulus conditions, using the ‘linalg.solve’ method in Numpy 1.6.2 (http://numpy.scipy.org) with Python 2.7.3 (http://www.python.org). Upon adding the ligand, we numerically solve the system of ODEs using the CVODE method in the SUNDIALS 2.5.0 package [38]. Thus, we focus on the typical experimental conditions that measure the response of the system to a sudden change of the ligand (TGF-β or TGF-β and BMP) concentration from zero to a saturating value (at time t = 0 hours) that is kept constant afterwards.

### Negative feedback network motifs

**Figure 2:**
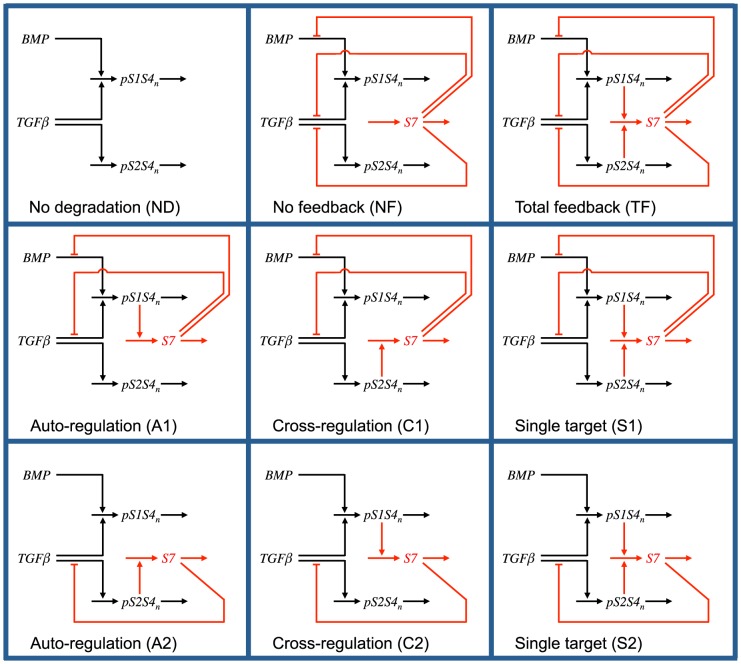
Schematic representation of the nine negative feedback loop network motifs. Horizontal arrows represent mass flow into and out of a species. Vertical arrows and flat-head lines terminating on a horizontal arrow denote activation or repression, respectively, of the targeted process. Processes common to all network motifs are drawn in black, while the unique negative feedback loop processes are colored red.

We have considered nine distinct network motifs for the Smad7-based negative feedback loop in the TGF-β signaling pathway, which are schematically represented in Figure 2. These include three unbiased network motifs where inhibition equally affects both R-Smad channels, denoted here by “no degradation” (ND), “no feedback” (NF), and “total feedback” (TF). The ND network motif captures a system lacking Smad7, eliminating Smad7-dependent ligand-induced degradation of active ligand-receptor complexes from the model. In the NF network motif, Smad7 is kept at a constant concentration, analogous to a system with saturated levels of Smad7. Therefore, the NF network motif provides inhibition without the Smad7 negative feedback loop. For the TF network motif, Smad7 expression is activated by, and inhibits, both R-Smad channels, which corresponds to the complete network represented in Figure 1.

The remaining six network motifs include biased inhibition of a single R-Smad channel. Two of them, denoted by A1 and A2, correspond to an auto-regulation negative feedback network motif, where Smad7 expression is activated by, and inhibits, the same R-Smad channel (Smad1 for A1 and Smad2 for A2). The C1 and C2 network motifs implement a cross-regulation negative feedback loop in which Smad7 expression is activated by the channel it does not inhibit. Finally, the network motifs denoted by S1 and S2 correspond to a negative feedback loop with a single target, where Smad7 expression is activated by both R-Smad channels, but inhibits only one of the channels. In all the cases, *RI_1B_* and *RI_1T_* are not separated in the examined variations of feedback motifs because Smad7 is supposed to bind to both of them in the same way. Specifically, both of them have the same type of L45 loop on the kinase domain, which determines the interaction with Smad7 through the L3 loop on the MH2 domain [39]. Since the kinetic parameters of Smad7 inhibition have not been fully characterized, based on this structural evidence, the most neutral assumption is to consider the same inhibitory kinetics for both of these type I receptors.

In order to implement each specific network motif, we eliminate particular Smad7-based processes by setting their rate constant values to zero (Table 2). In addition, the initial concentration of Smad7 is set to about 2,633 molecules for the NF network motif. This leads to a degradation rate of 0.395 min^−1^ for the active ligand-receptor complexes, as in Ref. [13], for the reference values for *k_20a,1T_, k_20a,1B_*, and *k_20a,2_* of Table 2. The initial Smad7 concentration for the other eight network motifs is zero. We include a representation of the model built with CellDesigner 4.2 [40] and the corresponding parameter values in File S1. This SBML file [41] corresponds to the TF network motif with its parameter set and initial conditions. The other cases are obtained by just setting the parameter values to those of Table 2.

**Table 2:** Parameter values of Smad7-related processes for the negative feedback network motifs.

Parameter	ND	NF	TF	A1	C1	S1	A2	C2	S2
*k_20a,1T_* (molec^−1^ min^−1^)	0	1.50×10^−4^	1.50×10^−4^	1.50×10^−4^	1.50×10^−4^	1.50×10^−4^	0	0	0
*k_20a,1B_* (molec^−1^ min^−1^)	0	1.50×10^−4^	1.50×10^−4^	1.50×10^−4^	1.50×10^−4^	1.50×10^−4^	0	0	0
*k_20a,2_* (molec^−1^ min^−1^)	0	1.50×10^−4^	1.50×10^−4^	0	0	0	1.50×10^−4^	1.50×10^−4^	1.50×10^−4^
*k_lip,1_* (molec min^−1^)	0	0	8.53×10^3^	8.53×10^3^	0	8.53×10^3^	0	8.53×10^3^	8.53×10^3^
*k_lip,2_* (molec min^−1^)	0	0	8.53×10^3^	0	8.53×10^3^	8.53×10^3^	8.53×10^3^	0	8.53×10^3^
*K_A,1_* (molec^−1^)	0	0	1.03×10^−6^	1.03×10^−6^	0	1.03×10^−6^	0	1.03×10^−6^	1.03×10^−6^
*K_A,2_* (molec^−1^)	0	0	1.03×10^−6^	0	1.03×10^−6^	1.03×10^−6^	1.03×10^−6^	0	1.03×10^−6^

Nonzero parameter values are from Ref. [25].

### Robustness analysis

The complexes *pS1S4_n_* and *pS2S4_n_* act as transcription factors to control the expression of hundreds of genes [5]. Thus, we focus on the different properties of the concentrations of these two species as the output of the model in our robustness analysis. Specifically, we investigate the following three properties: the peak species concentration (*m_p_*), the time at which the peak concentration is reached (*m_t_*), and the signal duration (*m_d_*) as defined in Ref. [42]. The peak species concentration, *m_p_*, is defined as 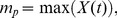
(1)which corresponds to the maximum of *X*(*t*), which is the concentration of *pS1S4_n_* or *pS2S4_n_* as a function of the simulation time *t*, with 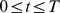. In the simulations, time *t* = 0 corresponds to the time at which the ligand is added and *t = T* corresponds to the total simulation time. We compute *m_t_* as 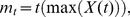
(2)which corresponds to the time at which *X*(*t*) reaches its maximum value *m_p_* within the simulation time. The final metric, *m_d_*, is given by 
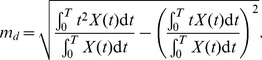
(3)


The signal duration metric captures the spread of *X*(*t*) about its peak [42]. In all three cases, we used a total simulation time *T* of 24 hours.

In order to assess the robustness of each network motif to parameter perturbation, we use the approach developed in Ref. [33] for the analysis of the IFN-γ induced JAK-STAT pathway. This method directly compares the parameter variation measure, defined by the logarithmic change in parameter values upon random perturbation, with the resulting metric output of the system. The parameter variation measure *M_i_* is defined as 
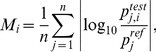
(4)where *n* is the number of nonzero parameters, 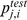 is the *j^th^* parameter of the *i^th^* test parameter set, and 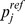 is the *j^th^* parameter in the reference parameter set [33]. For each parameter, we compute its test value by generating a random variable *x* with a uniform distribution within the range [−1, 1] and multiplying the reference parameter value by 10*^x^*. This is an adaptation of the sampling method used in Ref. [33], where the authors generate *x* from a standard normal distribution. Our approach adapts the parameter space approach used in the parameter optimization routine from Ref. [25]. Specifically, we generate the test parameter set within a parameter space defined as ±1 order of magnitude for each parameter, including the Smad7-associated parameters (*k_20a,1T_, k_20a,1B_, k_20a,2_, k_lip,1_, k_lip,2_*, *K_A,1_*, and *K_A,2_*). We generate 4,000 test parameter sets, as it is a sufficiently large number of samples for the results in this analysis to converge (see Text S1).

For each test parameter set *i*, we simulate the model and define its output as *C_i_*. The different cases studied correspond to diverse ligand stimulation conditions, properties of the output, and output species for each of the nine negative feedback network motifs. This leads to a total of 108 cases resulting from all the possible combinations of taking one item from each of the following four groups for each of the 4,000 test parameter sets:

- nine negative feedback network motifs: ND, NF, TF, A1, A2, C1, C2, S1, and S2;
- two ligand stimulation conditions: TGF-β alone and co-stimulation with TGF-β and BMP together;
- two species: *pS1S4_n_* and *pS2S4_n_*;
- three metrics: *m_p_*, *m_t_*, and *m_d_*.

Therefore, the output *C_i_* represents one of three metrics for one of the two species for one of the two ligand stimulation conditions for one of the nine negative feedback network motifs, where the subscript *i* indicates one of the 4000 test parameter sets. One particular case consists of, for instance, the study of the ND network motif with just TGF-β stimulation and assessing as output the *pS1S4_n_* species focusing on the *m_p_* metric for each test parameter set, yielding 4,000 values of *C_i_* for this case.

For each case, we define a single robustness measure *R* as the ratio of the variance of all 4,000 values of *M_i_* in Equation (4) with respect to that of *C_i_*. This is mathematically expressed as 
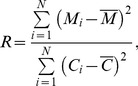
(5)where *N* is the number of parameter sets, equal to 4,000 here, and 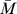 and 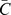 are the mean values of *M_i_* and *C_i_*, respectively. Larger values of *R* would indicate that the system is more robust to parameter perturbation, as the variance of the model output (*C*) is minimal compared to that of the parameter variation measure (*M*). We have calculated this robustness measure for all of the 108 cases.

### Sensitivity analysis

We have used a global sensitivity analysis to investigate how perturbations of individual parameters in the parameter space affect the model output [37]. Specifically, we performed a derivative-based global sensitivity analysis, which samples the effects of local parameter perturbation within the parameter space [43]. To estimate the effects of local parameter perturbation, we compute the scaled sensitivity coefficients [44] given by 
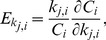
(6)where *k_j,i_* corresponds to the value of the *j^th^* parameter, *k_j_*, of sample *i* with its corresponding model output *C_i_*, defined as in the robustness analysis. To approximate the partial derivative, we evaluate the model with 0.5 percent perturbations of each parameter *k_j_* about its original value in sample *i* and calculate the finite central difference of the sensitivity metric [45].

For each parameter *k_j_*, we compute the local scaled sensitivity coefficients 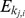 for the 4,000 parameter values by randomly sampling its value in the parameter space, as described in the previous section for the robustness analysis, and use them to obtain three sensitivity measures [43]. The first measure, denoted by 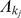, averages the absolute value of the local sensitivity coefficients and is given by 
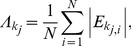
(7)where *N* is the number of parameter samples. The second measure, denoted by 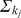, corresponds to the standard deviation of the local sensitivity coefficients and is given by 
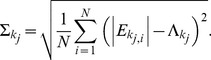
(8)


The third measure, 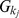, corresponds to the sum of the squares of the first two measures and is given by 
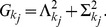
(9)


As the final measure combines the first two, we use 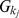 as the global sensitivity coefficient [43].

We implemented these robustness and sensitivity analyses using Python 2.7.3 (http://www.python.org), Numpy 1.6.2 (http://numpy.scipy.org), and Scipy 0.10.0 [46].

## Results

### Negative feedback network motifs' stimulation dynamics

**Figure 3:**
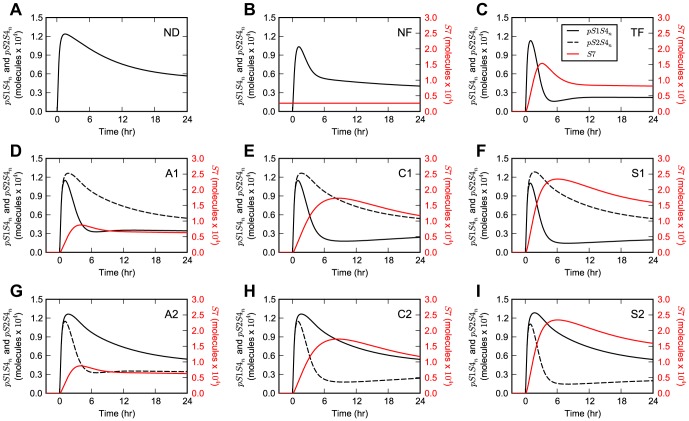
Model dynamics for stimulation with TGF-β. Simulation results upon single-ligand stimulation with TGF-β for the (**A**) ND, (**B**) NF, (**C**) TF, (**D**) A1, (**E**) C1, (**F**) S1, (**G**) A2, (**H**) C2, and (**I**) S2 network motifs. The simulation results of *pS1S4_n_*, *pS2S4_n_*, and *S7* are displayed as black solid lines, black dashed lines, and red solid lines, respectively.

The specific implementation of the negative feedback loop differentially affects the dynamics of the model upon TGF-β stimulation (Figure 3). Network motifs with unbiased inhibition of the R-Smad channels, namely ND, NF, and TF, provide both Smad1 and Smad2 channels with the same type of wiring upon single-ligand TGF-β stimulation. Therefore, when the parameters for both channels are the same as in our case, the results for the dynamics of nuclear pSmad1-Smad4 and pSmad2-Smad4 species are identical (Figures 3A, 3B and 3C). In contrast, in the case of network motifs with biased inhibition, i.e., those where Smad7 inhibits only one channel, such as A1, A2, C1, C2, S1, and S2, the system response varies between the two channels (Figures 3D, 3E, 3F, 3G, 3H, and 3I). In these network motifs, as Smad7 alternates its target between the Smad1 and Smad2 channels, the dynamic response of one R-Smad channel mirrors that of the other. For example, in the case of the auto-regulatory negative feedback network motifs, the *pS1S4_n_* response in the network motif A1 is identical to that of *pS2S4_n_* for the network motif A2 (Figures 3D and 3G).

**Figure 4:**
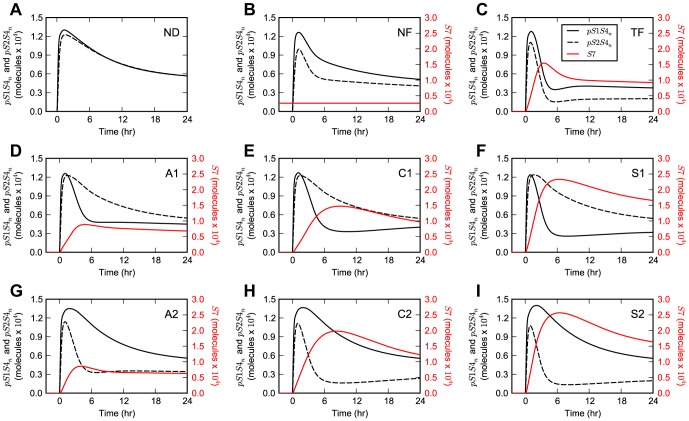
Model dynamics for stimulation with TGF-β and BMP. Simulation results upon co-stimulation with TGF-β and BMP for the (**A**) ND, (**B**) NF, (**C**) TF, (**D**) A1, (**E**) C1, (**F**) S1, (**G**) A2, (**H**) C2, and (**I**) S2 network motifs. The simulation results of *pS1S4_n_*, *pS2S4_n_*, and *S7* are displayed as black solid lines, black dashed lines, and red solid lines, respectively.

Upon co-stimulation of the system with both TGF-β and BMP ligands, the *pS1S4_n_* and *pS2S4_n_* signals are no longer identical for the ND, NF, and TF network motifs due to the additional activation of the Smad1 channel by BMP (Figures 4A, 4B, and 4C). In the case of the network motifs with biased architectures, namely A1, A2, C1, C2, S1, and S2, the system loses the mirrored behavior observed for R-Smad dynamics for stimulation with just the TGF-β ligand (Figures 4D, 4E, 4F, 4G, 4H, and 4I). Under both stimulation conditions, just with TGF-β or with TGF-β and BMP, the signaling species targeted by the negative feedback loop exhibits a pronounced transient response, while the other species exhibits a more sustained response.

### Robustness analysis

**Figure 5:**
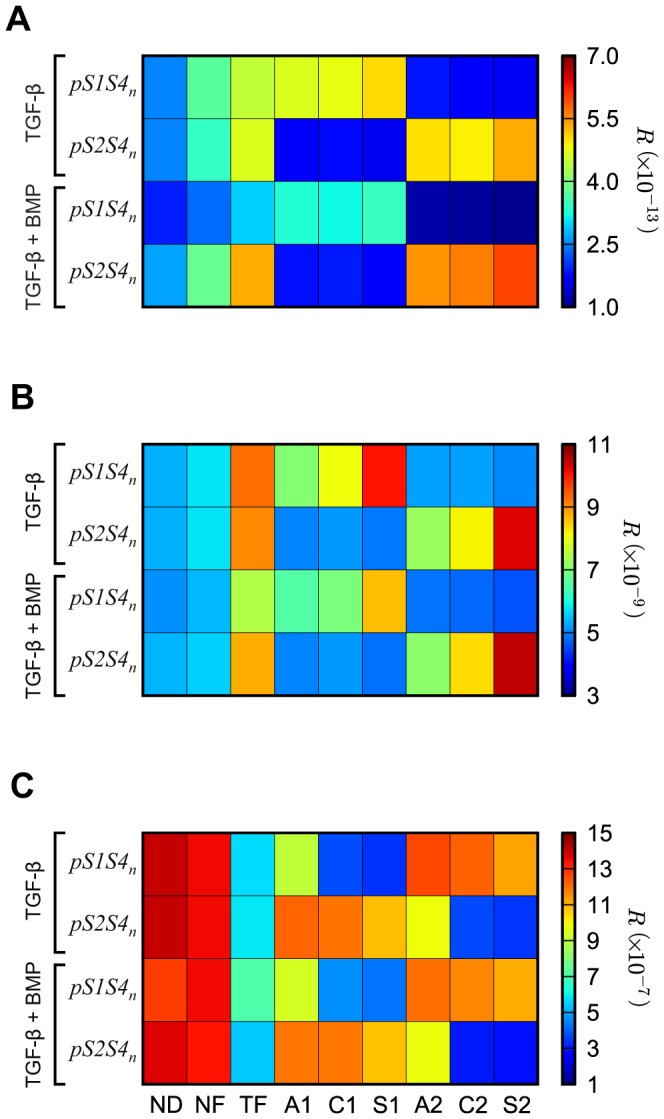
Robustness analysis. Each negative feedback network motif is assessed for its robustness by computing the value of the robustness measure *R* (Equation(5)) upon stimulation with TGF-β alone or together with BMP. To represent the model output, we use the (**A**) peak species concentration (*m_p_* metric; Equation(1)), (**B**) time of the peak species concentration (*m_t_* metric; Equation(2)), and (**C**) signal duration (*m_d_* metric; Equation(3)) as metrics of the *pS1S4_n_* and *pS2S4_n_* signal response.

Our analysis reveals distinct variability in robustness among the negative feedback network motifs (Figure 5). When considering the peak species concentration (*m_p_* metric; Equation(1)) as the system output, the ND network motif consistently displays low values of the robustness measure *R*, indicating low robustness to parameter perturbation under the two stimulation conditions studied, namely stimulation with TGF-β only and co-stimulation with TGF-β and BMP ligands, for both *pS1S4_n_* and *pS2S4_n_* species (Figure 5A). The NF and TF network motifs lead to higher values of *R*, consistent with an increase of the system robustness compared to that of the ND one. Note that the *R* values are slightly different between the *pS1S4n* and the *pS2S4n* cases with the stimulation of just TGF-β in the case of NF and TF network motifs because they have been computed with different realizations of the random values of each of the parameters in the 4000 test parameter sets. As the number of test parameter sets goes to infinity, the difference between these values should vanish.

The biased A1, A2, C1, C2, S1, and S2 network motifs exhibit a distinct pattern where the negative feedback loop increases the robustness of the species it targets in each case. For example, analyzing the *pS1S4_n_* maximum concentration as the model output upon stimulation with TGF-β (Figure 5A, top row), our results indicate that A1, C1, and S1 network motifs are more robust than those where the negative feedback loop inhibits the Smad2 channel (A2, C2, and S2) and *vice versa*. Stimulating the system with both TGF-β and BMP ligands results in lower values of the robustness measure for the Smad1 channel (*pS1S4_n_* species) for all network motifs when compared to stimulation with TGF-β alone, while the robustness of the Smad2 channel (*pS2S4_n_* species) increases, but is less affected by co-stimulation.

Considering other metrics, specifically the time at which the maximum concentration for the different species is reached (*m_t_* metric; Equation(2)), results in a qualitatively similar system robustness when compared to the *m_p_* metric for all network motifs and stimulation conditions (Figure 5B). In the case of the signal duration (*m_d_* metric; Equation(3)), the robustness analysis reveals a decreased robustness for the negative feedback loop, in contrast to the behavior observed for the *m_p_* and *m_t_* metrics (Figure 5C). In this case, the NF and ND network motifs display the highest robustness values, while robustness decreases for the network motifs in which the negative feedback loop inhibits the evaluated channel.

### Sensitivity analysis

**Figure 6:**
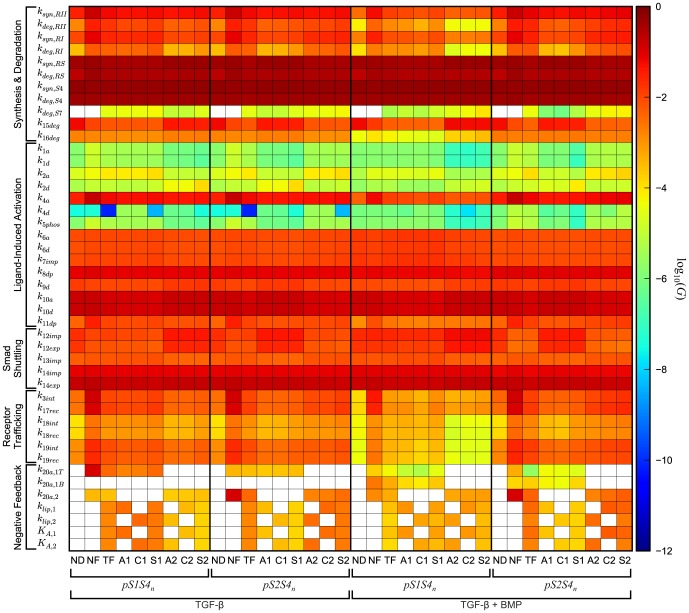
Sensitivity analysis for the peak species response metric *m_p_*. We compute the sensitivity coefficient 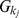 (Equation (9)) for each model parameter for the nine negative feedback network motifs upon stimulation with TGF-β alone or together with BMP. We use the metric *m_p_* (Equation(1)) to assess the signal response of *pS1S4_n_* and *pS2S4_n_*. Empty spaces (white rectangles) indicate a sensitivity coefficient equal to zero or a parameter value set to zero from Table 2.

The results of the sensitivity analysis are shown in Figure 6, Figure S1, and Figure S2 for the *m_p_*, *m_t_*, and *m_d_* metrics, respectively. For the peak species concentration metric *m_p_*, our analysis reveals a high network-motif-independent sensitivity, as given by a high value of the global sensitivity coefficients (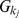 measure; Equation (9)), for multiple parameters, including the synthesis and degradation rate constants of the R-Smads (*k_syn,RS_* and *k_deg,RS_*) and Smad4 (*k_syn,S4_* and *k_deg,S4_*) (Figure 6). Additionally, the model is sensitive to the parameters governing the reversible association of nuclear phosphorylated R-Smad with Smad4 (*k_10a_* and *k_10d_*), nucleocytoplasmic shuttling of Smad4 (*k_14imp_* and *k_14exp_*), and degradation of nuclear phosphorylated R-Smads (*k_15deg_*) for all network motifs. The model's sensitivity for the Smad7-related parameter values, namely *k_20a,1T_*, *k_20a,1B_*, *k_20a,2_*, *k_lip,1_*, *k_lip,2_*, *K_A,1_*, and *K_A,2_*, is motif-dependent. Specifically, the model is more sensitive to these processes if the negative feedback loop inhibits the evaluated channel. For example, when stimulating the system with TGF-β alone and assessing the *pS1S4_n_* species as the output, this set of parameters has a higher sensitivity coefficient in the network motifs A1, C1, and S1 than in the network motifs A2, C2, and S2.

For the metric *m_t_* that characterizes the time at which the peak concentration is reached, the sensitivity analysis shows minimal differences in the sensitivity coefficients among all parameters (Figure S1). Nevertheless, it reveals a higher sensitivity for several groups of parameters in the network motifs where Smad7 does not inhibit activation of the evaluated species (e.g. A2, C2, and S2 network motifs are more sensitive than A1, C1, and S1 network motifs to perturbations of *k_10a_*, *k_10d_*, and *k_15deg_* parameters when evaluating the *pS1S4_n_* species). This effect is consistent with the results of the robustness analysis, in the sense that increases in the robustness measure *R* correspond with a decreased sensitivity coefficient.

The sensitivity analysis for the signal duration metric *m_d_* indicates that the model exhibits a higher sensitivity to parameter perturbation when the negative feedback loop inhibits the evaluated species (Figure S2), which is consistent with the robustness analysis results for this metric. This effect is most significant for the Smad7-related parameters *k_20a,1T_*, *k_20a,1B_*, *k_20a,2_*, *k_lip,1_*, *k_lip,2_*, *K_A,1_*, and *K_A,2_*.

## Discussion

The TGF-β signal transduction pathway is extensively regulated to effectively control multiple cellular responses [2], [6]. Here, we have characterized the effects of a key regulatory mechanism that controls the potential attenuation of the signal through a Smad7-dependent negative feedback loop. Our results show that variations in the network design of this negative feedback loop result in distinct dynamic behavior and response to parameter perturbation. Specifically, repression by the negative feedback loop results in a transient signal response, while the absence of this feedback results in a more sustained behavior. As regulation of gene expression by *pS1S4_n_* and *pS2S4_n_* is linked to this signaling property, requiring a prolonged signal to maximally activate transcription [47], negative feedback may be a central control mechanism for determining the long-term cellular response to ligand stimulation.

The robustness analysis for the *m_p_* and *m_t_* metrics revealed that the presence of the negative feedback loop correlated with increased robustness for the signaling channel it represses. However, our results also show that the three signaling metrics we examined do not respond identically to parameter perturbation. In particular, the robustness of the signal duration metric decreases for the species repressed by the negative feedback loop. With negative feedback, the repressed signal exhibits a transient response with shorter signal duration than the sustained response of the non-repressed signal. This type of transient response is characteristic of cancer cells [48]. Our results suggest that the shorter signal duration is more significantly affected by parameter perturbation than the greater signal duration exhibited by the species without negative feedback repression. Together, the three metrics show that the robustness of the system to parameter perturbation is dependent upon the specific negative feedback network motif, although in a different manner for the *m_p_* and *m_t_* metrics than the *m_d_* metric.

Similar to the robustness analysis, the sensitivity analysis shows varied results for the three signal metrics. Assessing the peak species concentration (*m_p_* metric) shows an increased sensitivity coefficient for the Smad7-based processes when the negative feedback loop represses the evaluated species. However, the robustness analysis for this metric shows an increased robustness with the addition of a negative feedback loop, revealing that, while the negative feedback loop itself may be sensitive to perturbation, its impact on the complete system results in additional robustness. With the *m_t_* metric, the specific negative feedback network motif has a minimal effect on the sensitivity coefficients for the majority of parameters. However, with several parameters (*k_syn,RS_*, *k_deg,RS_*, *k_10a_*, *k_10d_*, and *k_15deg_*), the sensitivity analysis shows as well that the negative feedback loop establishes resistance to perturbations by lowering the sensitivity coefficient compared with the network motifs lacking repression of the evaluated species.

These results, as a whole, show that the robustness and sensitivity properties depend both on the specific network motif and on the signal property of interest. This type of behavior is typically observed in other systems. For instance, in the classical example of bacterial chemotaxis [49], it was observed that steady-state behavior and adaptation time are not robust, while the precision of adaptation is robust to changes in protein concentrations. In our case, as in the case of bacterial chemotaxis, the adaptation time, or signal duration, is not a robust property. The general rule is that an increase in robustness against some perturbations will be counterbalanced by a decrease of robustness against other perturbations [50]. This rule is epitomized by the Bode integral formula, which represents conservation of sensitivity of a negative feedback system along the frequency axis [51], [52].

The available experimental data indicates that the negative feedback loop exists in several forms in different cell lines. In particular, bovine aortic endothelial cells exhibit an auto-regulatory negative feedback loop in which Smad7 is expressed through activation of the Smad1 channel and inhibits further activation of the same R-Smad channel [26]. In the mouse myoblast cell line C2C12, TGF-β does not activate BMP-responsive reporter genes [28], suggesting that the ligand induces transcriptional activity through the Smad2 channel. Results from experiments tracking the dynamics of R-Smad phosphorylation [27] and from computational modeling of the signaling behavior in these cells [25] suggest that Smad7 primarily inhibits activation of the Smad1 channel. Together, these findings provide evidence that C2C12 cells possess a cross-regulatory negative feedback loop in which Smad7 is expressed through the Smad2 channel, but inhibits the Smad1 channel when the pathway is activated with TGF-β. In contrast, human keratinocyte HaCaT cells exhibit a high basal concentration of Smad7 that is minimally affected upon treatment with TGF-β [29].

Our results are important because many components of the TGF-β signaling pathway are mutated, downregulated, or overexpressed in multiple diseases, such as the TGF-β receptors, R-Smads, Smad4, and Smad7 proteins in a variety of cancer types [3]. The sensitivity analysis captures the effects of these perturbations, quantifying how the model responds to variations in the pathway reactions. Indeed, our results from the sensitivity analysis identify several processes with high sensitivity coefficients, which are often dysregulated in cancer cells. For instance, missense mutations in the *Smad4* gene found in pancreatic cancer cells are associated with reduced nuclear translocation [53]. The model describes nuclear translocation of Smad4 with the rate constants *k_14imp_* and *k_14exp_*, both of which display among the highest sensitivity coefficients with the *m_p_* and *m_d_* metrics. Furthermore, missense mutations in the *Smad2* and *Smad4* genes occurring in colon and pancreatic cancer cells, respectively, have been reported to inhibit association of Smad2 with Smad4 [54]. The sensitivity analysis results show high sensitivity coefficients for the rate constants governing this process as well, where *k_10a_* and *k_10d_* regulate the nuclear association of phosphorylated Smad2 and Smad4. Notably, the sensitivity coefficients for *k_10a_* and *k_10d_* are dependent on the specific negative feedback network motif, which is most significantly observed with the *m_t_* metric, where network motifs lacking inhibition of the evaluated species display a higher sensitivity coefficient than those where the negative feedback loop inhibits the evaluated species. This correlation between model sensitivity and pathway mutation indicates that our analysis may be used to elucidate which processes are involved in the transition from normal to pathological states in a variety of cell types that exhibit the different negative feedback network motifs.

The results of the sensitivity analysis additionally provide a tool for determining novel targets in the pathway for therapeutic intervention. Potential therapeutic targets are defined as those where perturbations significantly affect the signaling response, such that administering treatment will maximally impact the dynamic behavior. By applying this analysis to the different negative feedback network motifs our results can be used to identify the therapeutic potential for targeting processes in a variety of cell types.

## Supporting Information

Figure S1 **Sensitivity analysis for the time of the peak species response metric** ***m_t_*** **.** We perform the same analysis described in the caption of Figure 6 using the *m_t_* metric (Equation (2)) to assess the signal response of *pS1S4_n_* and *pS2S4_n_*.

Figure S2 **Sensitivity analysis for the signal duration metric** ***m_d_*** **.** We perform the same analysis described in the caption of Figure 6 using the *m_d_* metric (Equation (3)) to assess the signal response of *pS1S4_n_* and *pS2S4_n_*.

Text S1 Supporting Text S1 file.

File S1 **SBML model representation of the TGF-β pathway.** The SBML file, built using CellDesigner 4.2, corresponds to the TF network motif with its parameter set and initial conditions.

## References

[1] Derynck R, Miyazono K (2008) The TGF-β Family. Cold Spring .Harbor, N.Y: Cold Sprint Harbor Laboratory Press. xiv, 1114 p.

[2] Massagué J (1998) TGF-β Signal Transduction. Annu Rev Biochem 67: 753–791. 9759503 10.1146/annurev.biochem.67.1.753

[3] Levy L, Hill CS (2006) Alterations in components of the TGF-β superfamily signaling pathways in human cancer. Cytokine Growth Factor Rev 17: 41–58. 16310402 10.1016/j.cytogfr.2005.09.009

[4] ten Dijke P, Arthur HM (2007) Extracellular control of TGFβ signalling in vascular development and disease. Nat Rev Mol Cell Biol 8: 857–869. 17895899 10.1038/nrm2262

[5] Shi Y, Massagué J (2003) Mechanisms of TGF-β Signaling from Cell Membrane to the Nucleus. Cell 113: 685–700. 12809600 10.1016/s0092-8674(03)00432-x

[6] Moustakas A, Heldin C-H (2009) The regulation of TGFβ signal transduction. Development 136: 3699–3714. 19855013 10.1242/dev.030338

[7] Hata A, Lagna G, Massagué J, Hemmati-Brivanlou A (1998) Smad6 inhibits BMP/Smad1 signaling by specifically competing with the Smad4 tumor suppressor. Genes Dev 12: 186–197. 9436979 10.1101/gad.12.2.186 PMC316444

[8] Nakao A, Afrakhte M, Morén A, Nakayama T, Christian JL, et al (1997) Identification of Smad7, a TGFβ-inducible antagonist of TGF-β signalling. Nature 389: 631–635. 9335507 10.1038/39369

[9] Goto K, Kamiya Y, Imamura T, Miyazono K, Miyazawa K (2007) Selective Inhibitory Effects Of Smad6 On Bone Morphogenetic Protein Type I Receptors. J Biol Chem 282: 20603–20611. 17493940 10.1074/jbc.M702100200

[10] Ebisawa T, Fukuchi M, Murakami G, Chiba T, Tanaka K, et al (2001) Smurf1 Interacts with Transforming Growth Factor-β Type I Receptor through Smad7 and Induces Receptor Degradation. J Biol Chem 276: 12477–12480. 11278251 10.1074/jbc.C100008200

[11] Kavsak P, Rasmussen RK, Causing CG, Bonni S, Zhu H, et al (2000) Smad7 Binds to Smurf2 to Form an E3 Ubiquitin Ligase that Targets the TGFβ Receptor for Degradation. Mol Cell 6: 1365–1375. 11163210 10.1016/s1097-2765(00)00134-9

[12] Wegner K, Bachmann A, Schad J-U, Lucarelli P, Sahle S, et al (2012) Dynamics and feedback loops in the transforming growth factor β signaling pathway. Biophys Chem 162: 22–34. 22284904 10.1016/j.bpc.2011.12.003

[13] Chung S-W, Miles FL, Sikes RA, Cooper CR, Farach-Carson MC, et al (2009) Quantitative Modeling and Analysis of the Transforming Growth Factor β Signaling Pathway. Biophys J 96: 1733–1750. 19254534 10.1016/j.bpj.2008.11.050 PMC2717289

[14] Clarke DC, Betterton MD, Liu X (2006) Systems theory of Smad signalling. IEE Proc-Syst Biol 153: 412–424. 10.1049/ip-syb:20050055 17186703

[15] Melke P, Jönsson H, Pardali E, ten Dijke P, Peterson C (2006) A Rate Equation Approach to Elucidate the Kinetics and Robustness of the TGF-β Pathway. Biophys J 91: 4368–4380. 17012329 10.1529/biophysj.105.080408 PMC1779910

[16] Paulsen M, Legewie S, Eils R, Karaulanov E, Niehrs C (2011) Negative feedback in the bone morphogenetic protein 4 (BMP4) synexpression group governs its dynamic signaling range and canalizes development. Proc Natl Acad Sci U S A 108: 10202–10207. 21633009 10.1073/pnas.1100179108 PMC3121836

[17] Schmierer B, Tournier AL, Bates PA, Hill CS (2008) Mathematical modeling identifies Smad nucleocytoplasmic shuttling as a dynamic signal-interpreting system. Proc Natl Acad Sci U S A 105: 6608–6613. 18443295 10.1073/pnas.0710134105 PMC2373357

[18] Vilar JMG, Jansen R, Sander C (2006) Signal Processing in the TGF-β Superfamily Ligand-Receptor Network. PLoS Comput Biol 2: e3. 16446785 10.1371/journal.pcbi.0020003 PMC1356091

[19] Vilar JMG, Saiz L (2011) Trafficking coordinate description of intracellular transport control of signaling networks. Biophys J 101: 2315–2323. 22098729 10.1016/j.bpj.2011.09.035 PMC3218327

[20] Celliere G, Fengos G, Herve M, Iber D (2011) Plasticity of TGF-beta signaling. BMC Syst Biol 5: 184. 22051045 10.1186/1752-0509-5-184 PMC3227652

[21] Zi Z, Feng Z, Chapnick DA, Dahl M, Deng D, et al (2011) Quantitative analysis of transient and sustained transforming growth factor-beta signaling dynamics. Mol Syst Biol 7: 492. 21613981 10.1038/msb.2011.22 PMC3130555

[22] Zi Z, Klipp E (2007) Constraint-Based Modeling and Kinetic Analysis of the Smad Dependent TGF-β Signaling Pathway. PloS One 2: e936. 17895977 10.1371/journal.pone.0000936 PMC1978528

[23] Zi Z, Chapnick DA, Liu X (2012) Dynamics of TGF-beta/Smad signaling. FEBS Lett 586: 1921–1928. 22710166 10.1016/j.febslet.2012.03.063 PMC4127320

[24] Ho J, Saiz L (2011) Computational Analysis of the TGF-Beta and BMP Signal Transduction Pathways. Biophys J 100: 164a.

[25] Nicklas D, Saiz L (2013) Computational modeling of Smad-mediated negative feedback and crosstalk in the TGF-β superfamily network. J R Soc Interface 10: 20130363. 23804438 10.1098/rsif.2013.0363 PMC3730684

[26] Valdimarsdottir G, Goumans M-J, Itoh F, Itoh S, Heldin C-H, et al (2006) Smad7 and protein phosphatase 1α are critical determinants in the duration of TGF-β/ALK1 signaling in endothelial cells. BMC Cell Biol 7: 16. 16571110 10.1186/1471-2121-7-16 PMC1479810

[27] Wrighton KH, Lin X, Yu PB, Feng X-H (2009) Transforming Growth Factor β Can Stimulate Smad1 Phosphorylation Independently of Bone Morphogenic Protein Receptors. J Biol Chem 284: 9755–9763. 19224917 10.1074/jbc.M809223200 PMC2665096

[28] Daly AC, Randall RA, Hill CS (2008) Transforming Growth Factor β-induced Smad1/5 Phosphorylation in Epithelial Cells is Mediated by Novel Receptor Complexes and Is Essential for Anchorage-Independent Growth. Mol Cell Biol 28: 6889–6902. 18794361 10.1128/MCB.01192-08 PMC2573298

[29] Edlund S, Lee SY, Grimsby S, Zhang S, Aspenström P, et al (2005) Interaction between Smad7 and β-Catenin: Importance for Transforming Growth Factor β-Induced Apoptosis. Mol Cell Biol 25: 1475–1488. 15684397 10.1128/MCB.25.4.1475-1488.2005 PMC548008

[30] Kitano H (2004) Biological robustness. Nat Rev Genet 5: 826–837. 15520792 10.1038/nrg1471

[31] Barkai N, Leibler S (1997) Robustness in simple biochemical networks. Nature 387: 913–917. 9202124 10.1038/43199

[32] Blüthgen N, Herzel H (2003) How robust are switches in intracellular signaling cascades? J Theor Biol 225: 293–300. 14604583 10.1016/s0022-5193(03)00247-9

[33] Zi Z-K, Sun Z-R (2005) Robustness Analysis of the IFN-γ Induced JAK-STAT Signaling Pathway. J Comput Sci & Technol 20: 491–495.

[34] Chen C, Cui J, Zhang W, Shen P (2007) Robustness analysis identifies the plausible model of the Bcl-2 apoptotic switch. FEBS Lett 581: 5143–5150. 17936275 10.1016/j.febslet.2007.09.063

[35] Zou X, Liu M, Pan Z (2008) Robustness analysis of EGFR signaling network with a multi-objective evolutionary algorithm. Biosystems 91: 245–261. 18053637 10.1016/j.biosystems.2007.10.001

[36] Milo R, Shen-Orr S, Itzkovitz S, Kashtan N, Chklovskii D, et al (2002) Network motifs: simple building blocks of complex networks. Science 298: 824–827. 12399590 10.1126/science.298.5594.824

[37] Zi Z (2011) Sensitivity analysis approaches applied to systems biology models. IET Syst Biol 5: 336–346. 22129029 10.1049/iet-syb.2011.0015

[38] Hindmarsh AC, Brown PN, Grant KE, Lee SL, Serban R, et al (2005) SUNDIALS: Suite of Nonlinear and Differential/Algebraic Equation Solvers. ACM Transactions on Mathematical Software 31: 363–396.

[39] Chen YG, Hata A, Lo RS, Wotton D, Shi Y, et al (1998) Determinants of specificity in TGF-beta signal transduction. Genes Dev 12: 2144–2152. 9679059 10.1101/gad.12.14.2144 PMC317013

[40] Funahashi A, Morohashi M, Kitano H (2003) CellDesigner: a process diagram editor for gene-regulatory and biochemical networks. BIOSILICO 1: 159–162.

[41] Hucka M, Finney A, Sauro HM, Bolouri H, Doyle JC, et al (2003) The systems biology markup language (SBML): a medium for representation and exchange of biochemical network models. Bioinformatics 19: 524–531. 12611808 10.1093/bioinformatics/btg015

[42] Heinrich R, Neel BG, Rapoport TA (2002) Mathematical Models of Protein Kinase Signal Transduction. Mol Cell 9: 957–970. 12049733 10.1016/s1097-2765(02)00528-2

[43] Kucherenko S, Rodriguez-Fernandez M, Pantelides C, Shah N (2009) Monte Carlo evaluation of derivative-based global sensitivity measures. Reliab Eng Syst Saf 94: 1135–1148.

[44] Varma A, Morbidelli M, Wu H (1999) Parametric sensitivity in chemical systems. Cambridge, U.K.; New YorkNY: Cambridge University Press. xvi, 342 p.

[45] Press WH, Teukolsky SA, Vetterling WT, Flannery BP (2007) Numerical recipes: the art of scientific computing. New York: Cambridge University Press. xxi, 1235 p.

[46] Jones E, Oliphant T, Peterson P, others (2001—) SciPy: Open source scientific tools for Python.

[47] Inman GJ, Nicolás FJ, Hill CS (2002) Nucleocytoplasmic Shuttling of Smads 2, 3, and 4 Permits Sensing of TGF-β Receptor Activity. Mol Cell 10: 283–294. 12191474 10.1016/s1097-2765(02)00585-3

[48] Nicolas FJ, Hill CS (2003) Attenuation of the TGF-beta-Smad signaling pathway in pancreatic tumor cells confers resistance to TGF-beta-induced growth arrest. Oncogene 22: 3698–3711. 12802277 10.1038/sj.onc.1206420

[49] Alon U, Surette MG, Barkai N, Leibler S (1999) Robustness in bacterial chemotaxis. Nature 397: 168–171. 9923680 10.1038/16483

[50] Kitano H (2007) Towards a theory of biological robustness. Mol Syst Biol 3: 137. 17882156 10.1038/msb4100179 PMC2013924

[51] Csete ME, Doyle JC (2002) Reverse engineering of biological complexity. Science 295: 1664–1669. 11872830 10.1126/science.1069981

[52] Bode HW (1945) Network analysis and feedback amplifier design. New York: D. Van Nostrand company, inc. 2 p. l., iii–xii, 551 p. p.

[53] Morén A, Itoh S, Moustakas A, Dijke P, Heldin CH (2000) Functional consequences of tumorigenic missense mutations in the amino-terminal domain of Smad4. Oncogene 19: 4396–4404. 10980615 10.1038/sj.onc.1203798

[54] Hata A, Lo RS, Wotton D, Lagna G, Massagué J (1997) Mutations increasing autoinhibition inactivate tumour suppressors Smad2 and Smad4. Nature 388: 82–87. 9214507 10.1038/40424

