# Correction: Characterization of Negative Feedback Network Motifs in the TGF-β Signaling Pathway

**DOI:** 10.1371/annotation/a6cc8c5a-716a-4772-b7ce-2fd203a5f3fb

**Published:** 2014-01-30

**Authors:** Daniel Nicklas, Leonor Saiz

There is an error at the beginning of the first sentence of the Abstract. {Chung, 2009 #1} is not the appropriate start of the Abstract. The correct sentence is: "The transforming growth factor-β (TGF-β) superfamily of cytokines plays a fundamental role in a wide variety of cellular processes, including growth, differentiation, apoptosis, and tissue homeostasis."

