# Supplementary figures and images for: Characterization of Negative Feedback Network Motifs in the TGF-β Signaling Pathway
Source: PLoS One. 2013 Dec 20;8(12):e83531. doi: 10.1371/journal.pone.0083531 (PMC3875243; doi:10.1371/journal.pone.0083531)

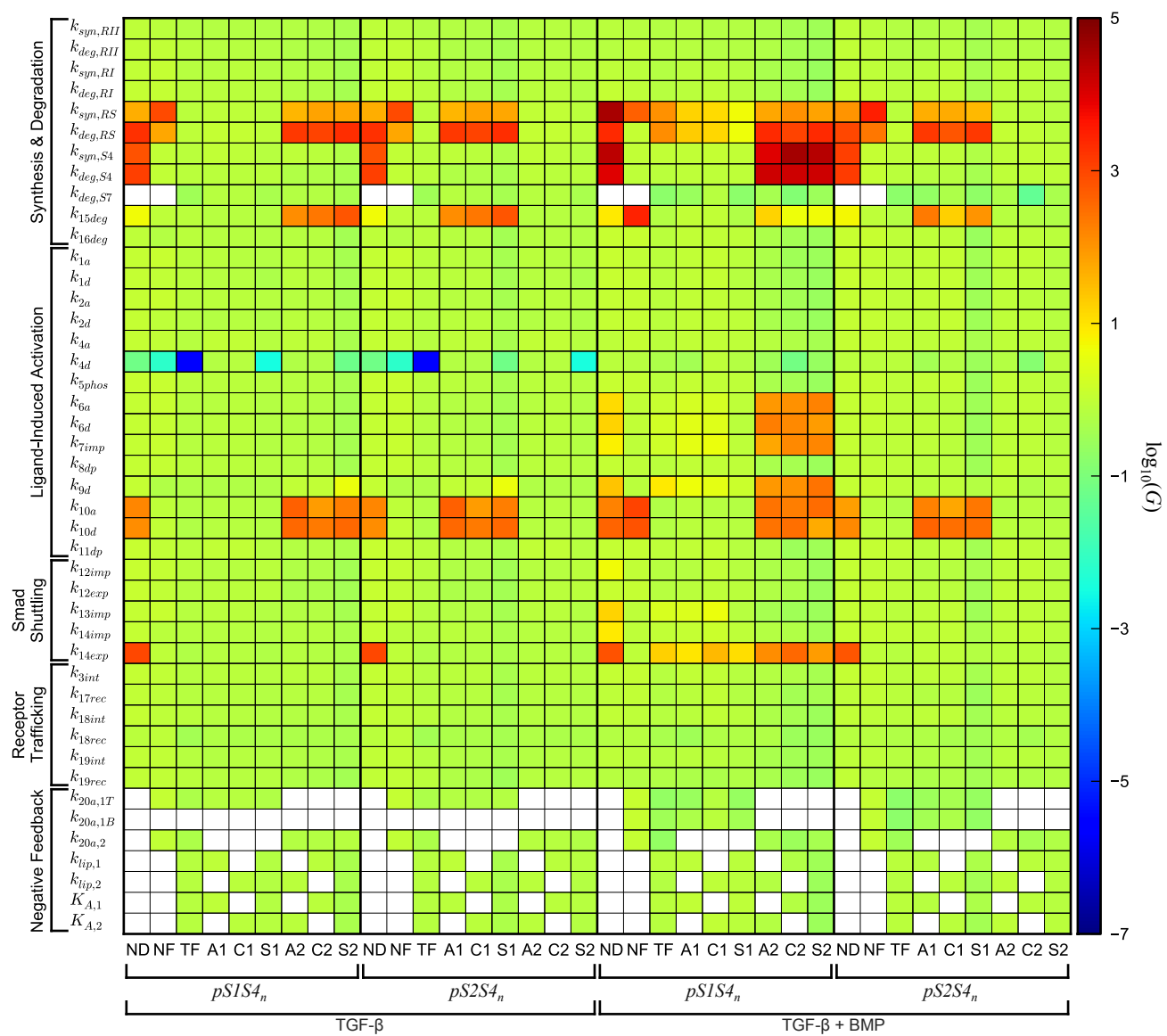

Figure S1

Supplement: Figure S1 — Sensitivity analysis for the time of the peak species response metric mt . We perform the same analysis described in the caption of Figure 6 using the mt metric (Equation (2)) to assess the signal response of pS1S4n and pS2S4n. [file pone.0083531.s001.pdf]

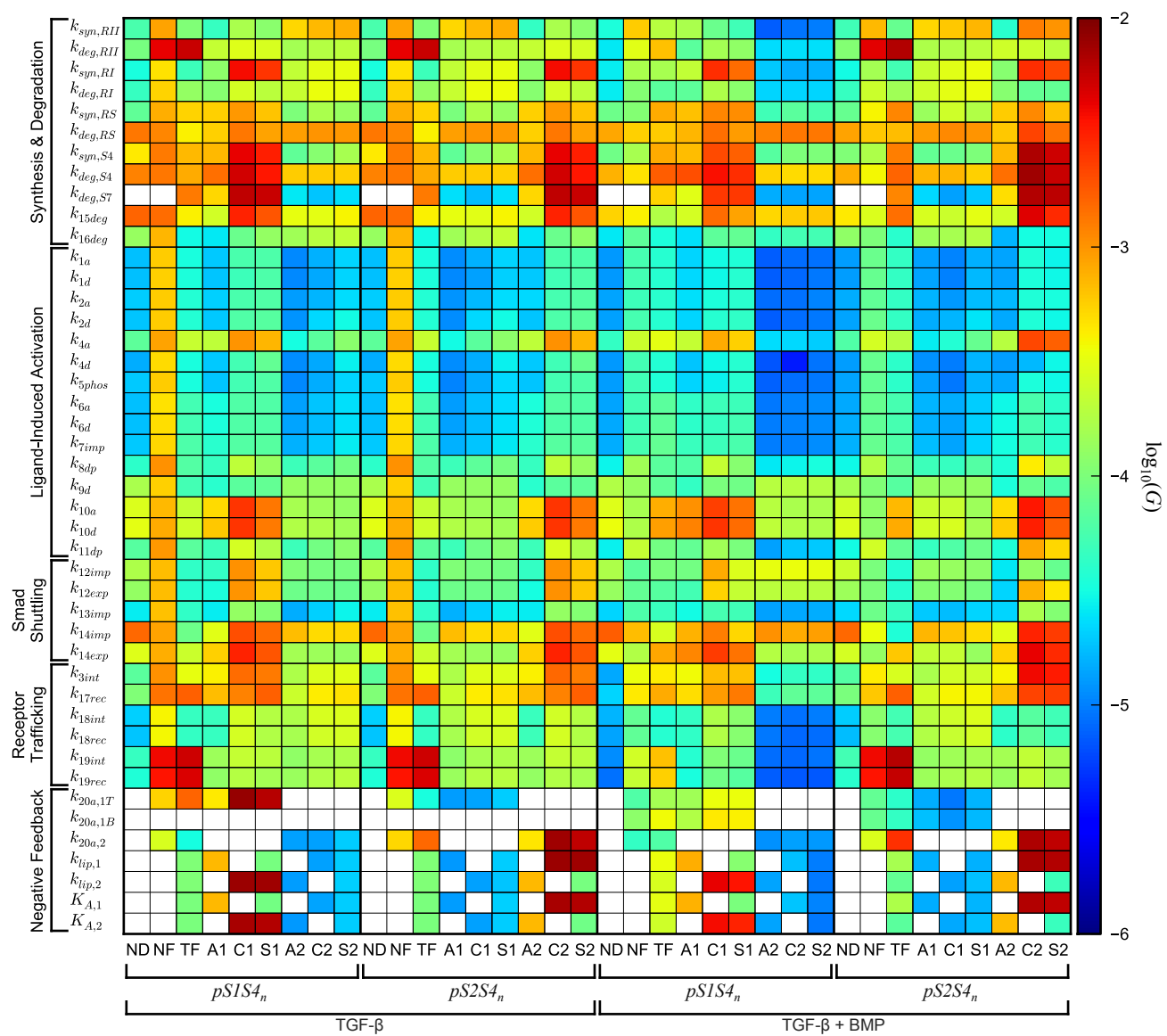

**Figure S2**

Supplement: Figure S2 — Sensitivity analysis for the signal duration metric md . We perform the same analysis described in the caption of Figure 6 using the md metric (Equation (3)) to assess the signal response of pS1S4n and pS2S4n. [file pone.0083531.s002.pdf]
