# Supplementary material for: Characterization of Negative Feedback Network Motifs in the TGF-β Signaling Pathway
Source: PLoS One. 2013 Dec 20;8(12):e83531. doi: 10.1371/journal.pone.0083531 (PMC3875243; doi:10.1371/journal.pone.0083531)
Supplement: Text S1 — Supporting Text S1 file. [file pone.0083531.s003.pdf]

Supporting Text S1

Robustness and sensitivity analysis convergence

To determine the number of samples required for the robustness and sensitivity analysis results to each converge near a single value, we compute the relative error between successive results with increasing sample size. Beginning with 100 samples, we compute the relative error, *S*, at 25-sample intervals, generating the following sequence:

$$S = \left\{ \frac{\left| a_k - a_{k-1} \right|}{a_{k-1}} \middle| a_k = y\left(25k\right), k \in \left[4,5,6,\ldots,N/25\right] \right\}, \qquad \text{(S1)}$$

where *y* is the result of the robustness or sensitivity analysis using the specified number of samples and *N* is the maximum number of samples (6,000 in Figure ST1). For the three metrics, we average the sequence of relative error values of all results. With the robustness analysis, this includes the value of *R* (Equation (5) of the main text) for all stimulation conditions, species, and negative feedback network motifs. With the sensitivity analysis, we use the sensitivity measure *G* (Equation (9) of the main text) for all stimulation conditions, species, negative feedback network motifs, and parameters. The results of this analysis for metrics m_p (Equation (1) of the main text), m_t (Equation (2) of the main text), and m_d (Equation (3) of the main text) indicate that the robustness and sensitivity analysis results each converge within 4,000 samples (Figure ST1).

Reduced ligand-receptor—Smad7 affinity

In the model, we have used the parameter values assigned for the HaCaT case in Ref. (Nicklas & Saiz, 2013) for each negative feedback network motif, with the exception of the ligand-receptor—Smad7 association rate constants ($k_{20a,1T}$, $k_{20a,1B}$, and $k_{20a,2}$). For these parameters, we

have used the value prior to optimization, i.e. 1.50×10^{-4} $molec^{-1}$ min^{-1}, instead of its optimized value for the HaCaT case of 2.96×10^{-5} $molec^{-1}$ min^{-1}. The higher value for this reaction rate provides increased contrast between the dynamics of each negative feedback network motif compared to the results with the lower value (Figure ST2). In Figure ST3A and Figure ST3B, we perform the same robustness and sensitivity analysis with the m_p metric described for Figure 5A and Figure 6, respectively, of the main text using the lower ligand-receptor—Smad7 affinity rate constant. These results demonstrate that the decreased value does not qualitatively affect the analysis results and are representative of the results using the m_t and m_d metrics.

References

Nicklas D, Saiz L (2013) Computational modeling of Smad-mediated negative feedback and crosstalk in the TGF- β superfamily network. J R Soc Interface 10: 20130363.

Figures

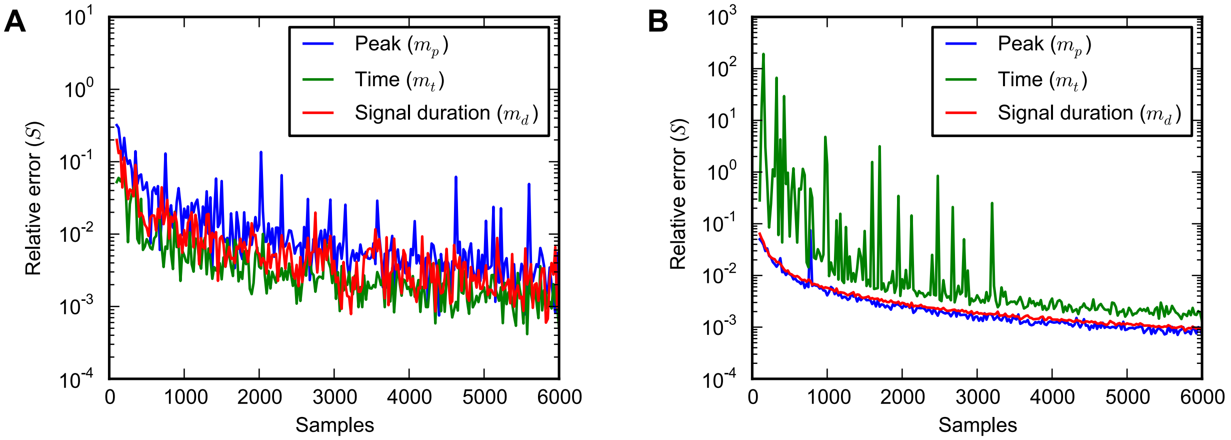

Figure ST1. Convergence of robustness and sensitivity analysis results. We compute the relative error (Equation S1) of the **(A)** robustness and **(B)** sensitivity analysis results with increasing number of samples for the peak, time, and signal duration metrics.

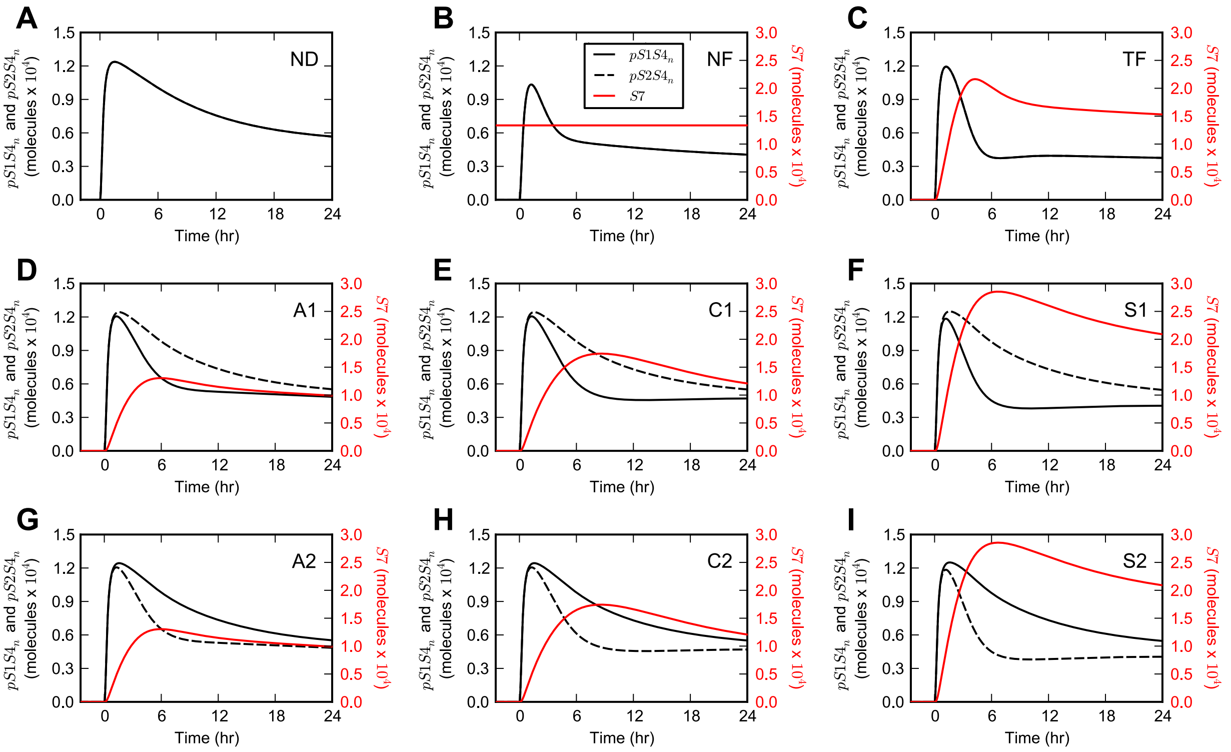

Figure ST2. Model dynamics for stimulation with TGF-β using a lower association rate constant for ligand-receptor—Smad7 binding. We perform the same analysis described for Figure 3 of the main text using 2.96×10^{-5} molec^{-1} min^{-1} for $k_{20a,1T}$, $k_{20a,1B}$, and $k_{20a,2}$.

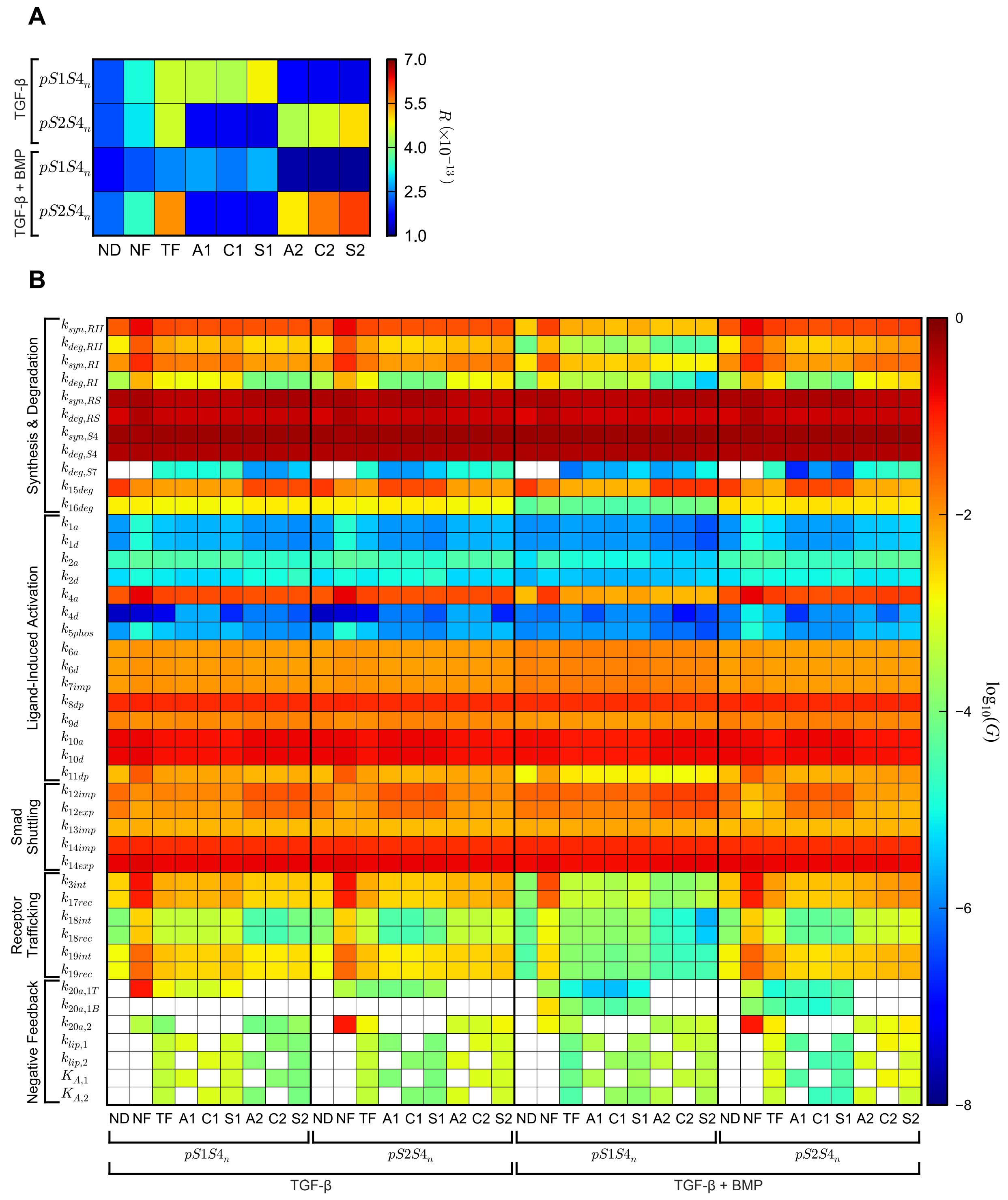

Figure ST3. Robustness and sensitivity analysis using a lower association rate constant for ligand-receptor—Smad7 binding. We perform the robustness and sensitivity analysis using 2.96×10^{-5} molec^{-1} min^{-1} for $k_{20a,1T}$, $k_{20a,1B}$, and $k_{20a,2}$. **(A)** Same analysis as Figure 5A of the main text with the lower rate constant values. **(B)** Same analysis as Figure 6 of the main text with the lower rate constant values.
